# Thalamocortical Projections Are Significantly Impaired in the R6/2 Mouse Model of Huntington’s Disease

**DOI:** 10.1523/ENEURO.0103-22.2022

**Published:** 2022-06-20

**Authors:** S. M. Holley, K. D. Oikonomou, C. M. Swift, L. Mohan, B. Matthews, O. Vega, G. Mkrtchyan, C. Cepeda, M. S. Levine

**Affiliations:** Intellectual and Developmental Disabilities Research Center, Semel Institute for Neuroscience and Human Behavior, University of California at Los Angeles, Los Angeles, CA 90095

**Keywords:** animal models, electrophysiology, Huntington’s disease, thalamocortical

## Abstract

As Huntington’s disease (HD) progresses, there is a significant loss of neurons in the striatum in addition to a distinct thinning of the cerebral cortex. Despite an early presence of sensorimotor deficits in patients with HD, electrophysiological studies designed to assess the integrity of thalamocortical circuits are sparse. Using the R6/2 mouse model of HD, we provide evidence of reduced connectivity between thalamic cells and their targeted cortical regions. Whole-cell patch clamp recordings from ventral anterolateral nucleus (VAL; motor) and ventral posteromedial nucleus (VPM; somatosensory) thalamic neurons in *ex vivo* brain slices of R6/2 and wild-type (WT) mice revealed that cells in both thalamic nuclei of R6/2 mice exhibited significant differences in passive and active cell membrane properties (smaller cell membrane capacitances, faster decay time constants and increased input resistances) compared with WT cells. Although only cells in the VPM of symptomatic R6/2 mice had more depolarized resting membrane potentials compared with WTs, cells in both nuclei displayed increased excitability in symptomatic, but not presymptomatic, R6/2 mice. Optical activation of VAL and VPM terminals elicited smaller magnitude current responses in cortical pyramidal neurons (CPNs) in both motor cortex (M1CTX) and somatosensory barrel cortex (BCTX) of symptomatic R6/2 mice compared with CPNs in WT mice. Furthermore, we observed a decrease in the frequency of thalamocortical excitatory quantal events in R6/2 BCTX CPNs, with no genotype-dependent differences in AMPA:NMDA response amplitude ratios. These data suggest there is a decrease in the transmission of thalamocortical information that is likely because of impaired neurotransmitter release.

## Significance Statement

The cellular electrophysiology of the thalamus has largely been underinvestigated in Huntington’s disease (HD). Here, we provide the first report in an HD mouse model that shows cells in both motor and somatosensory thalamic nuclei of R6/2 mice are impaired. There also exists a reduction in excitatory neurotransmission within the motor and somatosensory cortices and abated connectivity between the thalamus and cortex. These impairments would ultimately yield faulty processing of somatosensory and motor information that may contribute to the cognitive and motor deficits observed in patients with HD.

## Introduction

Huntington’s disease (HD) is a heritable and fatal neurodegenerative disorder characterized by impaired motor control in addition to cognitive and psychiatric disturbances (van Duijn et al., 2008; Bates et al., 2015; Snowden, 2017). HD is caused by an autosomal dominant mutation in at least one copy of the huntingtin (*HTT*) gene (The Huntington’s Disease Collaborative Research Group, 1993). In humans, the main histopathological feature of HD is the significant loss of neurons in the caudate nucleus and putamen (Vonsattel and DiFiglia, 1998; Reiner et al., 2013). However, as the disease advances, neurons in the cerebral cortex, hippocampus, hypothalamus and thalamus also are lost (Dom et al., 1976; Vonsattel, 2008; Waldvogel et al., 2015). Thus, HD can be thought of as a multisystem degenerative disorder where the decline of multiple brain regions contributes to its progressively debilitating phenotype.

Since the discovery of the mutated HD gene, numerous mouse models of HD have been created and characterized to better understand the neuropathological, synaptic and behavioral changes that occur in HD. One such model, the R6/2, is a transgenic mouse that expresses exon 1 of the human mutant gene and displays an aggressive phenotype with the onset of behavioral deficits appearing by five weeks and overt motor impairments occurring between 9 and 11 weeks (Mangiarini et al., 1996; Hickey et al., 2005). Although there is minimal cell loss in R6/2 brains, these mice exhibit significant brain atrophy and cellular degeneration (Stack et al., 2005). Additionally, aggregates of mutant huntingtin (mHTT) protein appear scattered throughout several brain regions including the striatum, cortex, thalamus, substantia nigra, as early as four weeks (Davies et al., 1997; Bates et al., 1998; Li et al., 1999; Morton et al., 2000). But, because there is significant neuronal loss in the striatum and cortex in HD patients, considerable emphasis has been placed on investigating these two brain regions in model mice (Raymond et al., 2011; Blumenstock and Dudanova, 2020; Barry et al., 2022; Cepeda and Levine, 2022) and few studies have focused on other brain regions such as the thalamus. Thalamic neurons make glutamatergic projections to striatum and cortex (Tsumoto, 1990; Jones, 2009; Smith et al., 2014), which function as an integral site for the integration and processing of motor and sensory information. Early reports in HD patients have shown decreases in the volume of specific thalamic nuclei at later stages of the disease, particularly mediodorsal (MD), centromedian/parafasicular (CM/Pf), intralaminar (IL), ventral anterolateral (VAL), and ventral posteromedial (VPM) nuclei (Dom et al., 1976; Heinsen et al., 1996, 1999; Kassubek et al., 2005). Synaptic studies designed to assess whether thalamocortical circuitry is altered in HD are limited despite the observation that early deficits in cognitive and sensorimotor performance tasks occur in patients and HD animal models before motor deficits (Boecker et al., 1999; Beglinger et al., 2010).

In the present study, we investigated the synaptic alterations in both the motor and somatosensory thalamocortical pathways in R6/2 mice. Using whole-cell patch clamp electrophysiology and optogenetics in *ex vivo* brain slices, we recorded cell membrane properties and synaptic inputs onto cortical pyramidal neurons (CPNs) in the primary motor (M1CTX) and somatosensory barrel (BCTX) cortices of ∼75-d-old wild-type (WT) and symptomatic R6/2 mice. The mouse BCTX is a specialized and well-organized region in the primary somatosensory cortex that is mapped to the individual vibrissae and is often used as a tool to investigate somatic and sensorimotor information processing (Petersen, 2007, 2019; Feldmeyer et al., 2013). However, few studies have reported synaptic alterations in the BCTX of HD model mice. Two reports demonstrated that R6/1 mice failed to learn a BCTX-dependent tactile discrimination task and displayed reduced plasticity in response to sensory deprivation following whisker removal (Cybulska-Klosowicz et al., 2004; Mazarakis et al., 2005). An additional recent study performed in early symptomatic YAC128 and zQ175 mice showed that cortical activity in barrel and sensory cortices is increased in these mice following forelimb stimulation (Sepers et al., 2022). Here, we report that passive and active cell membrane properties of CPNs in both M1CTX and BCTX are altered in R6/2 mice and these cells receive less thalamic inputs compared with CPNs from WT mice. Recordings from VAL and VPM thalamic cells in symptomatic R6/2 mice revealed that these cells displayed increased excitability, a reduction in spontaneous synaptic events and changes in cell membrane properties. We provide the first evidence in an HD mouse model showing significant alterations in passive and active membrane properties in both motor and somatosensory thalamic nuclei.

## Materials and Methods

### Mice

All experimental procedures were performed in accordance with the United States Public Health Service Guide for Care and Use of Laboratory Animals and were approved by the Institutional Animal Care and Use Committee at the University of California Los Angeles (UCLA). Mice were obtained from our breeding colony and every effort was made to minimize pain, discomfort, and the number of mice used. Animal housing conditions were maintained under a standard 12/12 h light/dark cycle (light cycle starting at 6 A.M. and ending at 6 P.M.) and at a temperature of 20–26°C. The animals had *ad libitum* access to food and water. All experiments were performed using the R6/2 mouse model of HD (CAG repeat length: 158 ± 1.8) and WT littermates. For these mice, WT male C57BL/6xCBA mice were crossed with WT female C57BL/6xCBA mice that had transplanted R6/2 ovaries (B6CBA-Tg(HDexon1)62Gpb/3J, RRID: IMSR_JAX:006494, The Jackson Laboratory). Both male and female mice were used for all experiments. We observed no consistent differences between sexes and the data were pooled. Total numbers of mice used for experiments were 45 WT (17 males, 28 females) and 48 hemizygous R6/2 mice (22 males, 26 females). In order to limit the number of mice used, wherever feasible, multiple experiments were performed using brain slices from the same mouse.

### Surgery

**Figure 1.**
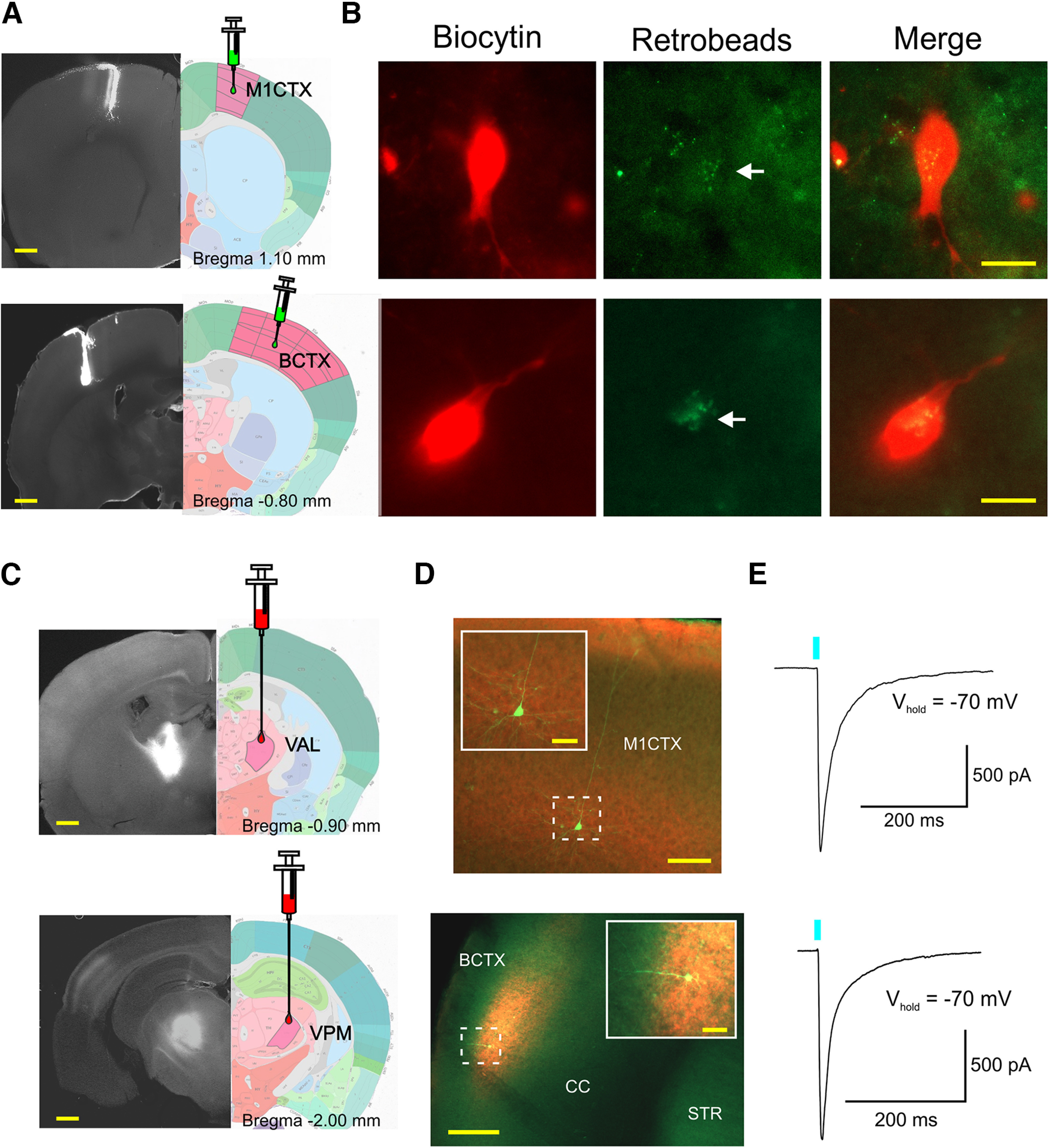
***A***, Fluorescence photomicrographs of retrobead-GFP injection sites (left panels) in the M1CTX (top image) and BCTX (bottom image) paired with single section images taken from the Allen Brain Atlas (right panels). Yellow scale bars: 500 μm. ***B***, Images of biocytin-filled recorded cells in the VAL (top images) and VPM (bottom images) identified with GFP retrobeads. White arrow (middle panels) points to visible retrobeads. Yellow scale bars: 10 μm. ***C***, Fluorescence photomicrographs (left) and single section images from the Allen Brain Atlas showing VAL (top) and VPM (bottom) AAV-ChR2-mCherry injection sites in VAL and VPM thalamic nuclei. ***D***, Biocytin-filled CPNs (green) recorded in Layers II/III of M1CTX (top) and Layer IV of BCTX (bottom). Insets show thalamic cell terminals expressing ChR2-mCherry surrounding recorded CPNs. Yellow scale bars: 200 and 20 μm (inset images) ***E***, Optically-evoked current responses of cells in ***D*** when stimulated with blue light (0.5 ms, 470 nm).

Mice were anesthetized with isoflurane, mounted into a stereotaxic frame and burr holes were drilled into the skull above selected injection sites. For experiments involving retrograde transport to thalamic neurons, surgeries were performed 4–25 d before recordings. Retrobeads-GFP (Lumafluor) were delivered bilaterally (0.25 μl/hemisphere) into the motor (M1CTX) or somatosensory BCTX at a rate of 0.1 μl/min and were localized to the injection site with minimal spread. For M1CTX, the stereotaxic injection coordinates were (relative to bregma): AP +1.5, ML ±1.5, and DV injections were consecutive, starting at −1.0 mm (1/2 volume) then −0.5 mm (1/2 volume) from the surface of the brain. BCTX injections coordinates were: AP −0.8, ML ±3.5, and DV −0.5 mm from brain surface, at an angle of 15° (Fig. 1*A*). For optogenetic experiments targeting selective activation of thalamocortical inputs, mice were injected bilaterally in the ventral anterior (VA)-ventral lateral (VL) motor thalamus complex (VAL) or the VPM sensory thalamic nuclei with AAV-CaMKII-hChR2(H134R)-mCherry (AAV2/1 serotype; University of Iowa Viral Vector Core Facility, Iowa City, IA, or UNC Vector Core, Chapel Hill, NC) at a rate of 0.1 μl/min. Coordinates for VAL injections were (relative to bregma): AP −0.8, ML ±1.0, DV −3.0 mm from the surface of the brain. VPM injection coordinates were AP −1.75, ML ±1.5, DV −3.3 mm from the surface of the brain (Fig. 1*C*). After each AAV injection (titer 3.0 × 10^12^ vg/ml), the needle remained in place for 5–7 min before retraction to avoid virus backflow. Mice were killed for electrophysiological experiments at approximately six to eight weeks after injection to ensure sufficient opsin expression.

### Brain slice preparation

R6/2 and WT mice were deeply anesthetized with isoflurane and perfused intracardially with an ice-cold sucrose slicing solution containing the following (in mM): 87 NaCl, 2.5 KCl, 0.5 CaCl_2_, 7 MgCl_2_, 1.25 NaH_2_PO_4_, 26 NaHCO_3_, and 75 sucrose, pH 7.2 (aerated with 95% O_2_/5% CO_2_, 290–300 mOsm/l). Brains were rapidly extracted following decapitation and placed in oxygenated, ice-cold sucrose slicing solution. Coronal slices containing the cortical or thalamic regions of interest were cut (300 μm) using a vibrating microtome (VT1000S; Leica Microsystems), then transferred to an incubating chamber containing artificial CSF (ACSF; in mM: 130 NaCl, 3 KCl, 1.25 NaH_2_PO_4_, 26 NaHCO_3_, 2 MgCl_2_, 2 CaCl_2_, and 10 glucose) oxygenated with 95% O_2_-5% CO_2_ (pH 7.2–7.4, 290–310 mOsm/l) at 32°C for 30 min. Slices continued to recover at room temperature for an additional 30 min before recordings.

### Whole-cell patch clamp electrophysiology

All recordings were performed at room temperature using an upright microscope (Olympus BX51WI) equipped with differential interference contrast optics and fluorescence imaging (Retiga Electro 1.4 Mp CCD monochromatic camera with Ocular 2.0 software, QImaging). GFP/eYFP- and mCherry-based fluorophores in cells and neuronal processes were visualized using light-emitting diodes (excitation at 488 and 585 nm, respectively). Whole-cell patch clamp recordings in voltage and current-clamp modes were obtained from CPNs and thalamic neurons using a MultiClamp 700B Amplifier (Molecular Devices) and the pCLAMP 10.3 acquisition software. Recordings in M1CTX were obtained from CPNs in Layers II/III and in Layer IV for BCTX CPNs, where ChR2-mCherry expressing thalamic cell terminals were highly visible. The patch pipette (3–5 MΩ) contained a cesium-based internal solution (in mM): 125 Cs-methanesulfonate, 4 NaCl, 1 MgCl_2_, 5 MgATP, 9 EGTA, 8 HEPES, 1 GTP-Tris, 10 phosphocreatine, and 0.1 leupeptin (pH 7.2 with CsOH, 270–280 mOsm/l) for voltage-clamp recordings or a K-gluconate-based solution containing (in mM): 112.5 K-gluconate, 4 NaCl, 17.5 KCl, 0.5 CaCl_2_, 1 MgCl_2_, 5 ATP (potassium salt), 1 NaGTP, 5 EGTA, 10 HEPES, pH 7.2 (270–280 mOsm/l) for current-clamp recordings. For some recordings, internal electrode solutions contained 0.2% biocytin for subsequent immunodetection of recorded cells. Cell membrane properties (capacitance, input resistance and time constant) were recorded within 5 min of breaking into the cell and at a holding potential of −70 mV. Rheobase measurements were obtained by injecting cells with depolarizing current pulses (5 ms) until firing was achieved. Input-output functions as a test for cellular excitability were recorded in response to a 1-s duration depolarizing current injection. Only recordings where the electrode access resistances were <30 MΩ at the start and end of experiments were used for analysis.

Spontaneous postsynaptic currents (sPSCs) in CPNs were recorded at room temperature in gap-free mode, filtered at 1 kHz during acquisition and digitized at 100 μs using Clampex 10.3. Spontaneous IPSCs (sIPSCs) were recorded at +10 mV in standard ACSF. Spontaneous EPSCs (sEPSCs) were recorded at −70 mV and in the presence of the GABA_A_ receptor antagonist picrotoxin (PTX, 100 μm). Miniature EPSCs (mEPSCs) were recorded in the presence of tetrodotoxin (TTX; 1 μm) to block action potentials.

For optogenetic experiments, thalamocortical terminals expressing channelrhodopsin (ChR2) were activated with a single light pulse (470 nm, 0.5 ms, 3 mW, CoolLED) delivered through the microscope epifluorescence illumination pathway (Fig. 1*D*,*E*). QX-314 Cl^–^ (4 mM) was included in the internal pipette solution to block activity-dependent Na^+^ channels. Evoked IPSCs in response to optical stimulation were recorded in voltage-clamp mode, at a holding potential of +10 mV and in ACSF. In the same cells, optically-evoked EPSCs (oEPSCs) were recorded at −70 mV and in the presence of PTX (100 μm) and TTX (1 μm). For recording optically-evoked NMDA currents, cells were held at +40 mV and 6-cyano-7-nitroquinoxaline-2,3-dione (CNQX; 10 μm) was added to block AMPA receptors. In order to record quantal release of excitatory neurotransmitters, thalamocortical terminals were optically stimulated in an extracellular bath solution containing 4 mM Sr^2+^ and 0 Ca^2+^ (Goda and Stevens, 1994; McGarry and Carter, 2017). Quantal events recorded in 20 sweeps were measured to determine the average amount of neurotransmitter released per cell within 1 s following stimulation.

Following recordings, brain slices containing biocytin-filled cells were fixed in 4% PFA for 24 h. Slices were then washed with 0.1 m PBS, permeabilized with 1% Triton overnight at 4°C, and incubated for 2 h with Alexa Fluor 594-conjugated streptavidin (1:1000, ThermoFisher Scientific) at room temperature. Fluorescent images of biocytin-filled recorded cells were obtained using a Zeiss confocal ApoTome equipped with 20× and 40× objectives.

The following drug reagents were obtained from Tocris Bioscience/Bio-Techne: BIC, CNQX, QX314 Cl^–^. Picrotoxin and strontium chloride were obtained from Sigma-Aldrich and TTX from Calbiochem/MilliporeSigma. All drug stocks were made using double-distilled water and working solutions containing picrotoxin and strontium chloride were made in ACSF.

### Data analysis and statistics

Data are reported as mean ± SEM. Statistical analyses were performed using Student’s *t* tests or Mann–Whitney rank-sum tests for two group comparisons and appropriately designed two-way ANOVAs followed by Bonferroni *post hoc* tests for multiple group comparisons. Differences were considered statistically significant if *p* <0.05. sPSCs were analyzed off-line using the automatic detection protocol within the Mini Analysis Program (Synaptosoft) and subsequently checked manually for accuracy. Event analyses were performed blind to genotype. Measurements and analyses of individual postsynaptic responses evoked by optical stimulation and all current clamp measurements were performed using Clampfit 10.3.

## Results

### Thalamic neurons in both the VAL and VPM nuclei of symptomatic R6/2 exhibit changes in membrane properties and increased excitability

Since neurons in various brain regions tend to be more excitable in symptomatic HD mice (Klapstein et al., 2001; Cummings et al., 2009; Heikkinen et al., 2012), we examined whether thalamic neurons in VAL and VPM nuclei were altered in symptomatic R6/2 mice (75 d old: age range 64–83 d; 14 WT and 14 R6/2). At this age, R6/2 mice display decreased mobility and performance, significant weight loss, increased tremors, gait abnormalities and sensory-gating deficits (Mangiarini et al., 1996; Hickey et al., 2005; Stack et al., 2005; Menalled et al., 2009). For comparison, we also recorded in voltage clamp mode from VAL and VPM thalamic neurons in younger mice (21 d old: age range 18–25 d; 14 WT and 14 R6/2) absent of motor impairment. In order to target the thalamic cells that project specifically to motor and somatosensory cortices we injected GFP-labeled retrobeads into the M1CTX or BCTX of mice 4–25 d before recordings (Fig. 1*A*). Retrobeads are taken up by axon terminals and transported retrogradely to cell bodies (Katz and Iarovici, 1990). Expression of retrobeads in thalamic neurons was rapid in younger mice (Mean 6.8 ± 0.2 d) but required more time in older mice (mean 17.2 ± 0.7 d), regardless of genotype. Retrobeads were visible in thalamic cells from coronal brain slices using fluorescent imaging (Fig. 1*B*). Passive and active cell membrane properties were recorded from VAL and VPM cells containing retrobeads and are summarized in Table 1. Compared with WT cells, R6/2 cells in both nuclei exhibited significantly smaller membrane capacitance (*p* = 0.011 and *p* < 0.001 for VAL and VPM, respectively) and increased input resistance (*p* < 0.001 and *p* = 0.004 for VAL and VPM, respectively) at 75 d. Only VPM cells at this age had significantly faster membrane time constants (*p* = 0.157 and *p* < 0.001 for VAL and VPM, respectively). In presymptomatic 21-d-old R6/2 mice membrane capacitance of VAL cells was significantly reduced compared with WT cells (*p* < 0.001) and membrane input resistance was increased in VPM cells (*p* = 0.03) from R6/2 mice. These data suggest that in symptomatic R6/2 mice, thalamic neurons in both nuclei exhibit changes in membrane properties, some of which are present as early as 21 d. Age-related changes also are evident in WT VAL cells as membrane capacitance is significantly decreased with age (*p* = 0.004, 21 vs 75 d) and may contribute to genotype-dependent changes observed in R6/2 mice at the symptomatic age.

**Table 1:** Passive and active membrane properties of VAL and VPM neurons in 21-d-old (presymptomatic) and 75-d-old (symptomatic) R6/2 and WT mice

	Capacitance (pF)	Input resistance (MΩ)	Time constant (ms)	RMP (mV)	Rheobase (pA)
VAL					
21 d					
WT-VAL (*n* = 16)	173.2 ± 10.8	328.1 ± 31.2	4.2 ± 0.3	−64.5 ± 1.4	566.3 ± 21.9
R6/2-VAL (*n* = 14)	125.2 ± 7.7***	334.0 ± 54.2	3.0 ± 0.3***	−64.5 ± 1.5	396.4 ± 35.3**
75 d					
WT-VAL (*n* = 16)	136.9 ± 8.8^††^	245.3 ± 36.6	3.0 ± 0.2^†††^	−62.1 ± 0.8	360 ± 30.7^†††^
R6/2-VAL (*n* = 20)	107.1 ± 5.9*	460.2 ± 40.3***^§^	2.5 ± 0.1	−63.4 ± 1.4	360 ± 29.2
VPM					
21 d					
WT-VPM (*n* = 13)	175.2 ± 14.4	203.0 ± 34.1	3.8 ± 0.03	−63.1 ± 1.1	439.6 ± 52.9
R6/2-VPM (*n* = 16)	167.0 ± 13.6	351.1 ± 46.5*	3.7 ± 0.3	−64.1 ± 1.2	463.1 ± 40.9
75 d					
WT-VPM (*n* = 17)	151.1 ± 13.7	213.8 ± 29.5	3.2 ± 0.3	−64.2 ± 0.9	365.3 ± 38.7
R6/2-VPM (*n* = 21)	79.2 ± 4.7***^§§^	387.8 ± 50.3**	1.9 ± 0.1***^§§§^	−59.3 ± 1.4**	235.0 ± 29.9*^§§§^

Statistical significance was determined using two-way ANOVAs followed by Bonferroni *post hoc* tests. Symbols indicate the following: *, **, *** represent genotype-dependent statistically significant differences between cells in each age group and where *p* < 0.05, *p* < 0.01, and *p* < 0.001, respectively; ^††^, ^†††^ represent statistical significance when comparing age-dependent differences in WT cells in each nucleus and where *p* < 0.01 and *p* < 0.001, respectively; ^§^, ^§§^, ^§§§^ represent statistical significance when comparing age-dependent differences in R6/2 cells within each nucleus, and where *p* < 0.05, *p* < 0.01, and *p* < 0.001, respectively.

**Figure 2.**
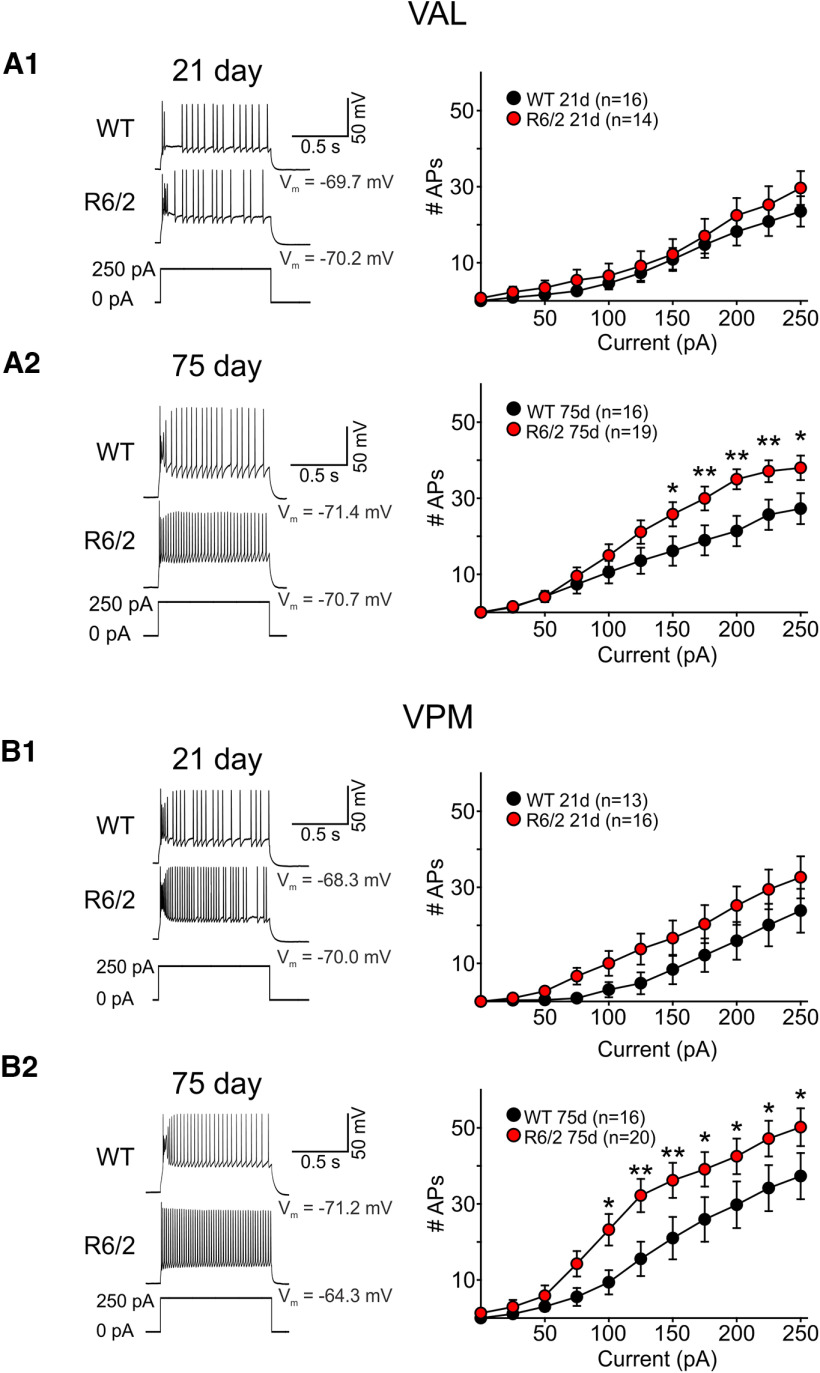
***A1***, ***A2***, Sample recorded excitability traces from WT and R6/2 VAL neurons (left) and summary plot of input-output mean number of spikes at 21 and 75 d. ***B1***, ***B2***, Sample recorded excitability traces from WT and R6/2 VPM neurons (left) and summary plot of input-output mean number of spikes at 21 and 75 d. Thalamic neurons fired action potentials when injected with a suprathreshold current pulse. Injected current pulses (1 s) started from the resting membrane potential (Vm) and were increased in steps of 25 pA. Statistical significance was determined using two-way ANOVAs followed by Bonferroni *post hoc* tests; *p < 0.05, **p < 0.01.

We next measured the intrinsic excitability of thalamic neurons while in current clamp mode (Fig. 2). At rest, only VPM cells in symptomatic 75-d-old R6/2 mice had RMPs that were significantly more depolarized compared with WTs (*p* = 0.004; Table 1). Rheobase measurements also were reduced in R6/2 VPM cells at this age (*p* = 0.017; Table 1). While we observed no significant differences in the RMPs of VAL cells at 21 d, rheobase measurements were significantly lower in R6/2s (*p* = 0.001). Regional comparisons of RMPs in R6/2 mice show that VPM cells were significantly more depolarized than VAL cells (*p* = 0.029) but in 75-d-old mice only (*p* = 0.793 for VAL 75 vs 21 d). Incidentally, the rheobase measurements in cells from 21- and 75-d-old mice also differed in WT VAL cells, where the cells at 21 d were observed to have a significantly higher rheobase (*p* = 0.001). Corroborating the differences observed in RMP measurements, input-output distributions for VPM cells from 75-d R6/2 mice also significantly differed. R6/2 VPM cells elicited more action potentials in response to increasing depolarizing current injections (1-s pulse) thus indicating that VPM cells in symptomatic mice are more excitable compared with cells from WTs (*p* = 0.003; Fig. 2). Similarly, we observed VAL cells in 75-d-old R6/2 mice fired more action potentials than WT cells in response to a sustained current pulse (*p* < 0.001). Input-output mean number of spikes in 21-d-old mice did not significantly differ between thalamic cells in R6/2 and WT mice, although there was a slight trend (*p* = 0.156) for increased action potential firing in VPM cells. There also was a trend for regional differences when comparisons between input-output mean number of spikes in symptomatic R6/2 mice were performed (*p* = 0.058). Greater number of action potentials were evoked in response to current injections in VPM cells compared with VAL cells in 75-d-old R6/2 mice. Bonferroni *post hoc* analysis indicated there was a significantly greater number of induced action potentials in VPM cells with 125-, 150-, 225-, and 250-pA current injections (*p* = 0.02, 0.028, 0.023, and 0.011, respectively). Taken together, these data suggest cells in both the VAL and VPM of symptomatic R6/2 mice are more excitable; however, since VPM neurons in symptomatic R6/2 mice also are more depolarized they may be more compromised than VAL neurons at this stage of the disease.

**Figure 3.**
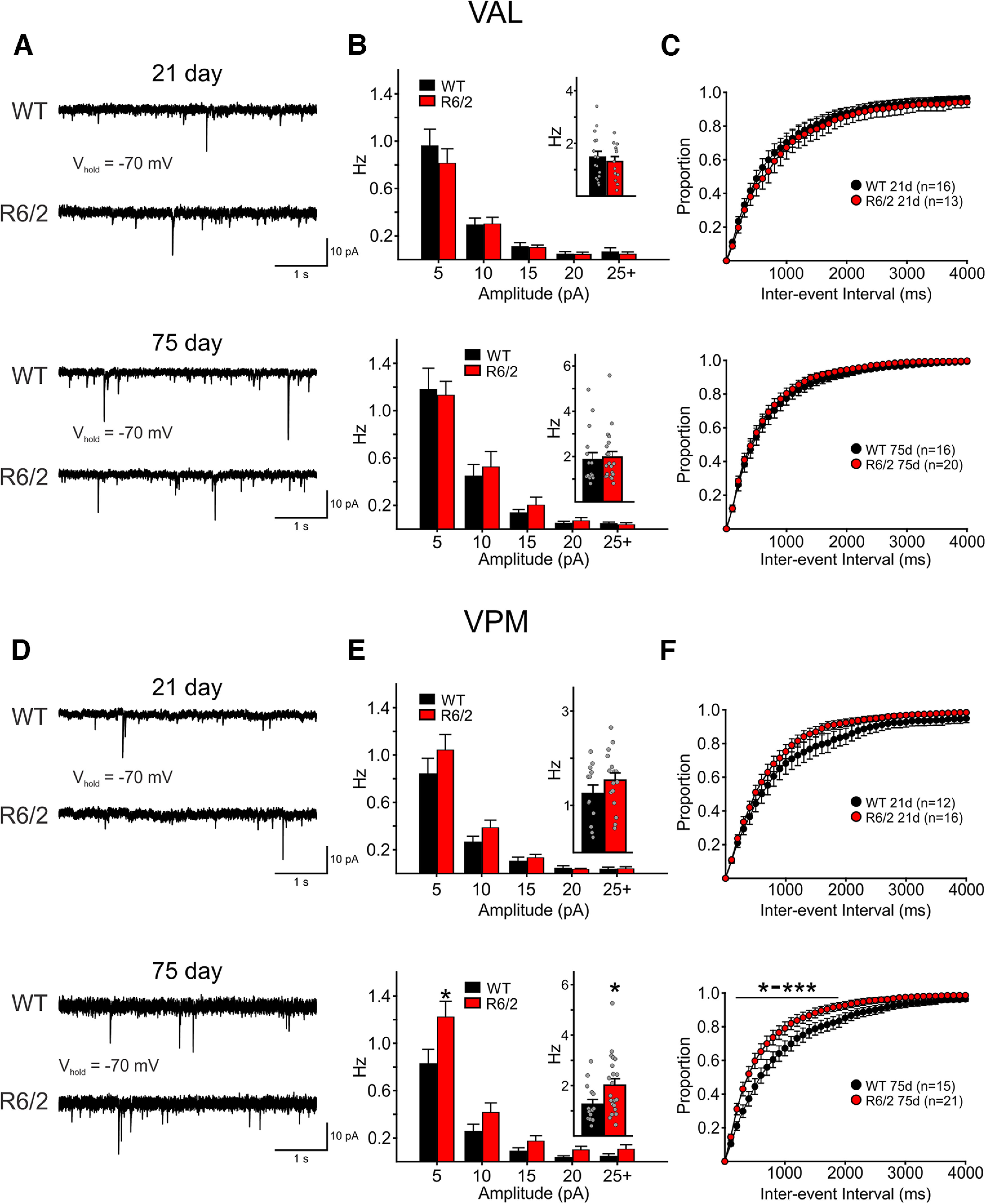
Sample raw traces of sPSCs (SPCs) recorded in 21-d (top) and 75-d (bottom) VAL (***A***) and VPM (***D***) neurons from WT and R6/2 mice. In all recordings, cells were held at −70 mV. Average SPC amplitude-frequency distribution plots (5-pA bins) of recordings in VAL (***B***) and VPM (***E***) cells. Mean SPC frequencies are shown as insets. SPC interevent interval distributions for VAL (***C***) and VPM (***F***) neurons showing the proportion of events recorded in different time intervals. Statistical significance was determined using two-way repeated measures ANOVAs followed by Bonferroni *post hoc* tests and where * to *** represent *p*-values of 0.05–0.001.

The sPSCs also were recorded in VAL and VPM cells from 21-d-old (*n* = 16 WT VAL, *n* = 14 R6/2 VAL; *n* = 12 WT VPM, *n* = 16 R6/2 VPM) and 75-d-old R6/2 and WT mice (*n* = 16 WT VAL, *n* = 20 R6/2 VAL; *n* = 15 WT VPM, *n* = 21 R6/2 VPM; Fig. 3). The frequencies of sPSCs (Vm held at –70 mV) were similar between genotypes in all age groups in both thalamic regions except in VPM cells at 75 d. Here, average sPSC frequency in cells from R6/2 mice was increased compared with the frequency in cells from WTs (*p* = 0.024, Mann–Whitney rank-sum test; Fig. 3*E*, bottom). This significant increase in sPSC frequency occurred primarily in events of 5–10 pA. The average amplitude of sPSCs was similar between genotypes at all ages in both regions (data not shown). Interevent interval distributions also show that sPSC frequencies in VPM cells from R6/2 mice were increased compared with WTs (*p* = 0.05 to *p* < 0.001 for intervals of 200–1900 ms; Fig. 3*F*). These data suggest that an increase in spontaneous synaptic inputs onto VPM cells in 75-d-old R6/2 mice may contribute to the increased excitability exhibited by these cells. However, it remains unclear from what cell types and from which brain regions these increased inputs originate.

### sEPSCs and sIPSCs are decreased in R6/2 CPNs compared with those of WTs

**Figure 4.**
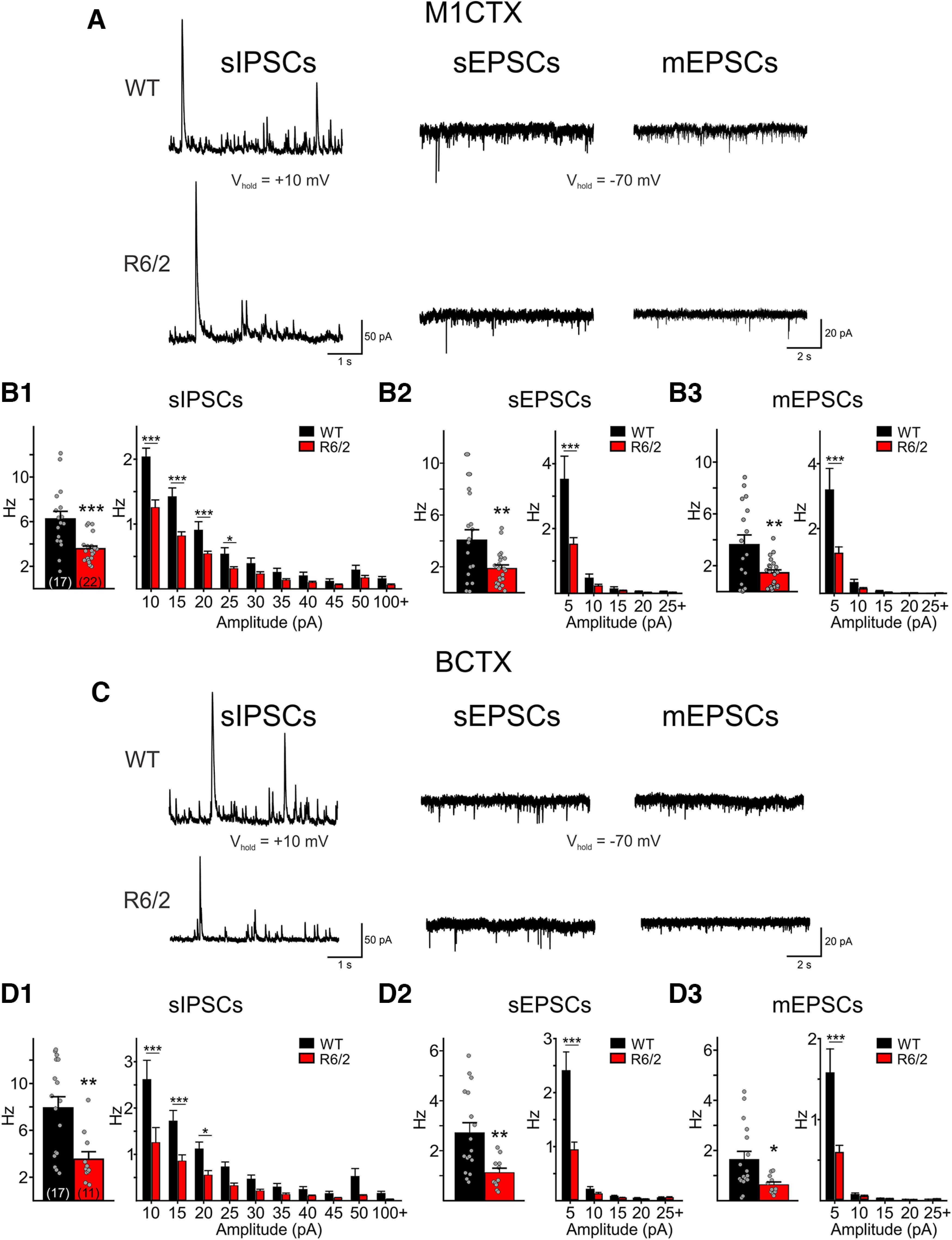
***A***, Sample raw traces of sIPSCs, sEPSCs, and mEPSCs recorded in M1CTX CPNs from a WT and a symptomatic R6/2 mouse. Summary of average frequencies of sIPSCs (***B1***), sEPSCs (***B2***), and mEPSCs (***B3***) in all recorded M1CTX CPNs (left). The number of recorded cells is shown in parentheses. Summary of average amplitude-frequency distribution plots (5-pA bins) are shown on the right. ***C***, Sample raw traces of sIPSCs, sEPSCs, and mEPSCs recorded in a BCTX CPN from a WT and a symptomatic R6/2 mouse. Summary of average frequencies of sIPSCs (***D1***), sEPSCs (***D2***), and mEPSCs (***D3***) in all recorded BCTX CPNs (left). Average amplitude-frequency distribution plots (5-pA bins) are shown on the right. Significant differences between genotypes were determined using Student’s *t* tests and two-way repeated measures ANOVAs followed by Bonferroni *post hoc* tests; *p < 0.05, **p < 0.01, and ***p < 0.001.

To better understand circuitry communication, we examined the frequencies of sIPSCs and sEPSCs in Layers II/III M1CTX CPNs in symptomatic 75-d-old R6/2 (age range 70–89 d; 11 mice) and WT mice (70–93 d; 8 mice; Fig. 4*A*,*B1–B3*). We also recorded sIPSCs and sEPSCs in somatosensory Layer IV BCTX CPNs to provide regional comparisons (age range 67–99 d; *n* = 9 mice each for R6/2 and WT; Fig. 4*C*,*D1–D3*). Cell membrane properties of M1CTX and BCTX CPNs were recorded at a holding potential of −70 mV and are summarized in Table 2.

**Table 2:** Cell membrane properties of M1CTX and BCTX neurons in symptomatic 75-d-old R6/2 and WT mice

	Capacitance (pF)	Input resistance (MΩ)	Time constant (ms)
M1CTX			
WT (*n* = 17)	247.2 ± 15.2	135.9 ± 12.4	4.4 ± 0.3
R6/2 (*n* = 22)	152.8 ± 10.9***	293.8 ± 28.9***	2.8 ± 0.2***
			
BCTX			
WT (*n* = 17)	193.5 ± 18.4	192.7 ± 23.1	4.1 ± 0.4
R6/2 (*n* = 11)	114.8 ± 9.3**	299.1 ± 27.1**	2.4 ± 0.2**

Statistical significance was determined using Student’s *t* tests. Symbols indicate the following: **, *** represent genotype-dependent statistically significant differences between cells in each group and where *p* < 0.01 and *p* < 0.001, respectively.

CPN cell membrane properties in both cortical regions differed in R6/2 mice compared with WTs in similar ways. Membrane capacitance was reduced, input resistance was higher and time constants were faster in R6/2 CPNs. The frequencies of sIPSCs and sEPSCs were significantly reduced in M1CTX and BCTX CPNs in R6/2 mice compared with WTs (sIPSCs: *p* < 0.001 for R6/2 vs WT M1CTX CPNs and *p* = 0.0024 for R6/2 vs WT BCTX CPNs; sEPSCs: *p* = 0.0066 for R6/2 vs WT M1CTX CPNs and *p* = 0.0049 for R6/2 vs WT BCTX CPNs). In both cortical regions and for both sEPSCs and sIPSCs, significant differences were observed primarily in lower amplitude events (Fig. 4*B1*,*B2* and *D1*,*D2*, right plots). mEPSCs were recorded following the addition of TTX (1 μm). Frequencies of mEPSCs in M1CTX and BCTX CPNs were also lower in R6/2 mice compared with WTs (*p* = 0.0042 for R6/2 vs WT M1CTX CPNs and *p* = 0.0195 for R6/2 vs WT BCTX CPNs; Fig. 4*B3*,*D3*). These data suggest that spontaneous excitatory and inhibitory synaptic transmission is reduced in both motor and somatosensory CPNs of symptomatic R6/2 mice.

### Thalamocortical synaptic connectivity is reduced in R6/2 mice

**Figure 5.**
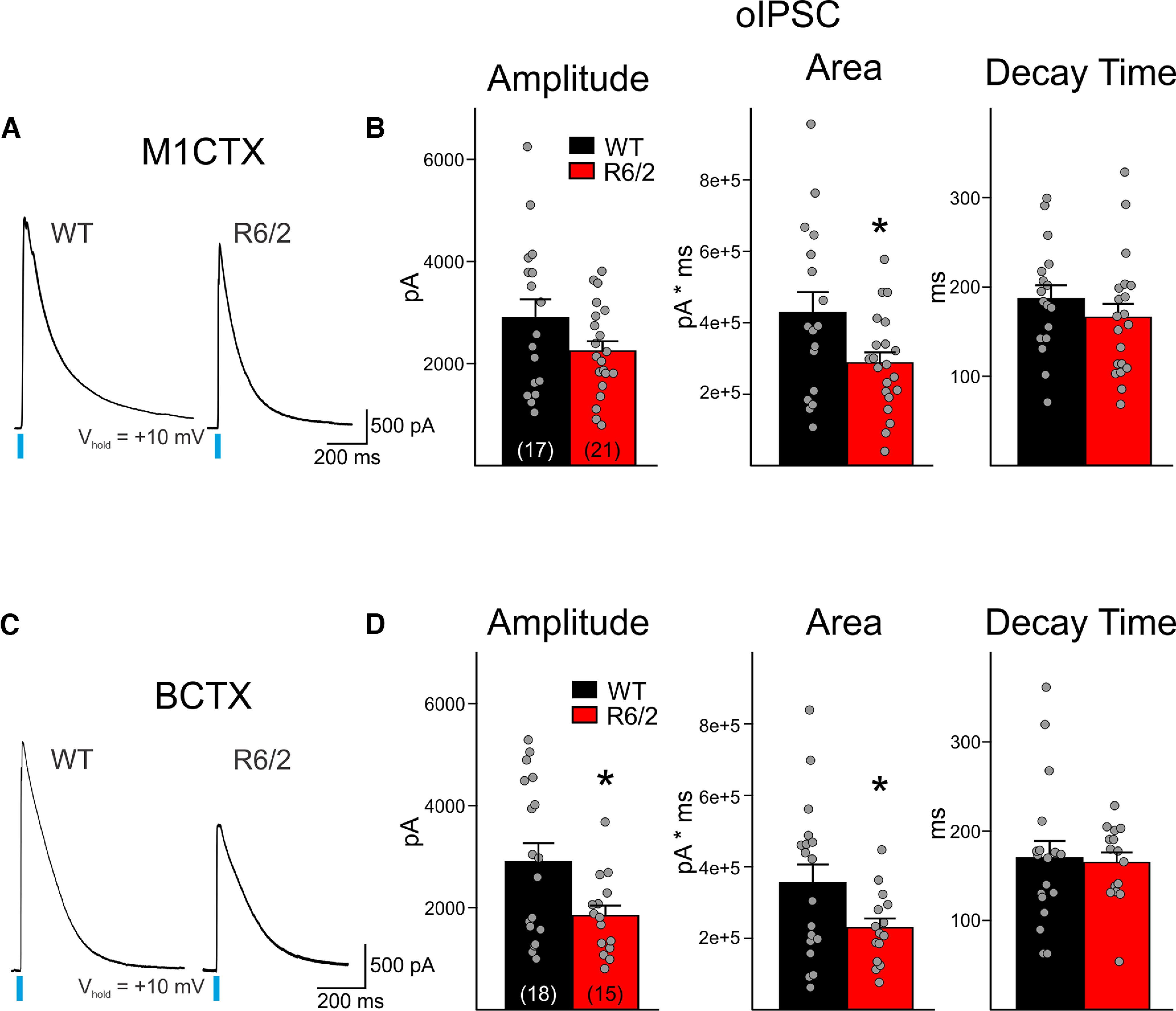
***A***, Sample traces of evoked thalamus-driven disynaptic or multisynaptic IPSCs (oIPSC; 3 sweeps, averaged) in WT and R6/2 M1CTX CPNs following optical activation of thalamic inputs (0.5 ms, 470 nm, 3 mW). The evoked current responses were the result of optical stimulation of a disynaptic network, since responses could be blocked with the addition of TTX (data not shown). ***B***, Summary graphs showing M1CTX oIPSC response properties. ***C***, Sample traces of evoked oIPSCs (3 sweeps, averaged) in WT and R6/2 BCTX CPNs following optical activation of thalamic inputs (0.5 ms, 470 nm, 3 mW). ***D***, Summary graphs showing BCTX oIPSC response properties. Statistical significance was determined with Student’s *t* tests; *p < 0.05.

Although the lower frequency of sEPSCs would suggest that CPNs in R6/2 mice receive reduced synaptic inputs, it is unclear which synaptic contacts are altered. Thalamocortical projections from the motor thalamic nuclei (VA/VL or VAL) synapse onto CPNs that reside in multiple layers, although the majority of projections terminate onto Layer II. Thalamocortical projections from the sensory thalamus (VPM) mostly target deep layer CPNs (Layers IV and V). To dissect out whether alterations of thalamocortical projections contribute to the reduced excitatory inputs in CPNs, we used optogenetics to specifically target thalamocortical projections originating from motor and sensory thalamic nuclei. Channelrhodopsin (CaMKII-ChR2-mCherry) was injected in the VAL or VPM thalamus and optically-evoked current responses were recorded in M1CTX or BCTX CPNs in symptomatic R6/2 and WT mice (Fig. 1*C–E*). For Layers II/III M1CTX, where VAL thalamocortical axons terminate, optical activation produced large IPSC responses in CPNs (Fig. 5*A*,*B*). This optically-evoked IPSC (oIPSC) was the result of activation of disynaptic or multisynaptic pathways since these responses could be abolished when action potential propagation was prevented following application of TTX (data not shown). The amplitudes of evoked oIPSCs in R6/2 M1CTX CPNs were slightly reduced and only the area of these was significantly different from responses recorded in WTs (*p* = 0.0301). In R6/2 BCTX CPNs (Layer IV), we also observed smaller evoked oIPSCs, and both amplitude and area were significantly reduced (*p* = 0.0210, for amplitude in R6/2 vs WTs and *p* = 0.0486 for area; Fig. 5*C*,*D*). No genotype-dependent differences in the evoked oIPSC response decay times were observed for CPNs in either brain region.

**Figure 6.**
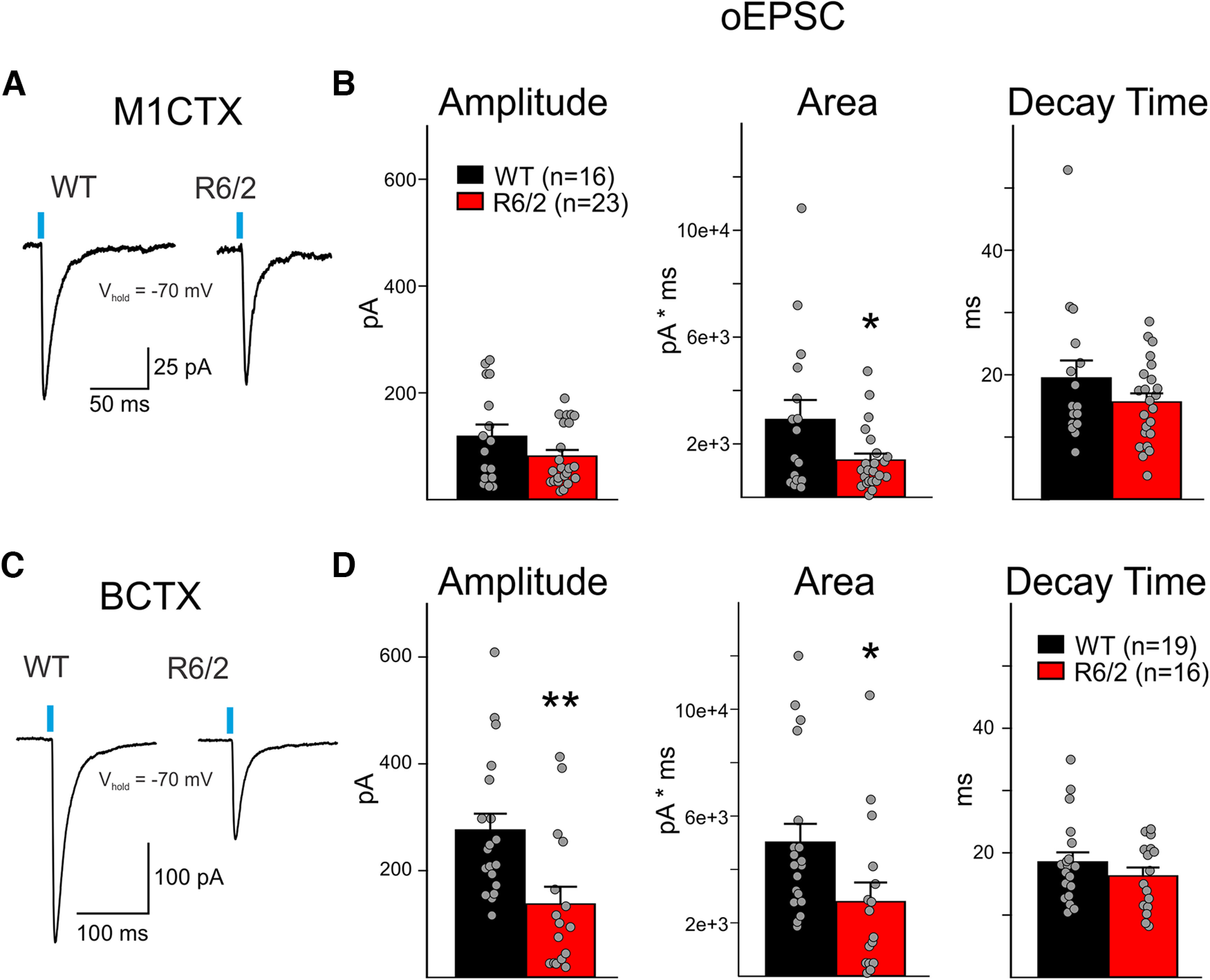
***A***, Sample traces of evoked monosynaptic EPSCs (oEPSC; 3 sweeps, averaged) in WT and R6/2 M1CTX CPNs following optical activation of thalamic inputs (0.5 ms, 470 nm, 3 mW). ***B***, Summary graphs showing M1CTX oEPSC response properties. ***C***, Sample traces of evoked monosynaptic oEPSCs (3 sweeps, averaged) in WT and R6/2 BCTX CPNs following optical activation of thalamic inputs (0.5 ms, 470 nm, 3 mW). ***D***, Summary graphs showing BCTX oEPSC response properties. Statistical significance was determined with Student’s *t* tests; *p < 0.05, **p < 0.01.

We then recorded in M1CTX and BCTX CPNs monosynaptic, oEPSCs, following the addition of the GABA_A_ receptor antagonist, picrotoxin, and TTX (Fig. 6*A–D*). In the M1CTX, oEPSCs in R6/2 CPNs displayed significantly reduced areas compared with responses observed in WTs (*p* = 0.0301; Fig. 6*A*,*B*). There also was a trend for reduced amplitude in oEPSC responses in R6/2 M1CTX CPNs compared with WTs (*p* = 0.1139). In BCTX CPNs, oEPSC responses to activation of thalamic terminals displayed significantly smaller amplitudes and areas (*p* = 0.004 for amplitude and *p* = 0.0324 for area in R6/2 vs WTs; Fig. 6*C*,*D*). For both cortical regions, no significant genotype-dependent differences occurred in the decay times of the optically-evoked responses. These results suggest reduced connectivity between thalamus and both motor and somatosensory areas in symptomatic R6/2 mice.

### The frequency of neurotransmitter release from thalamocortical terminals is decreased in R6/2 CPNs

**Figure 7.**
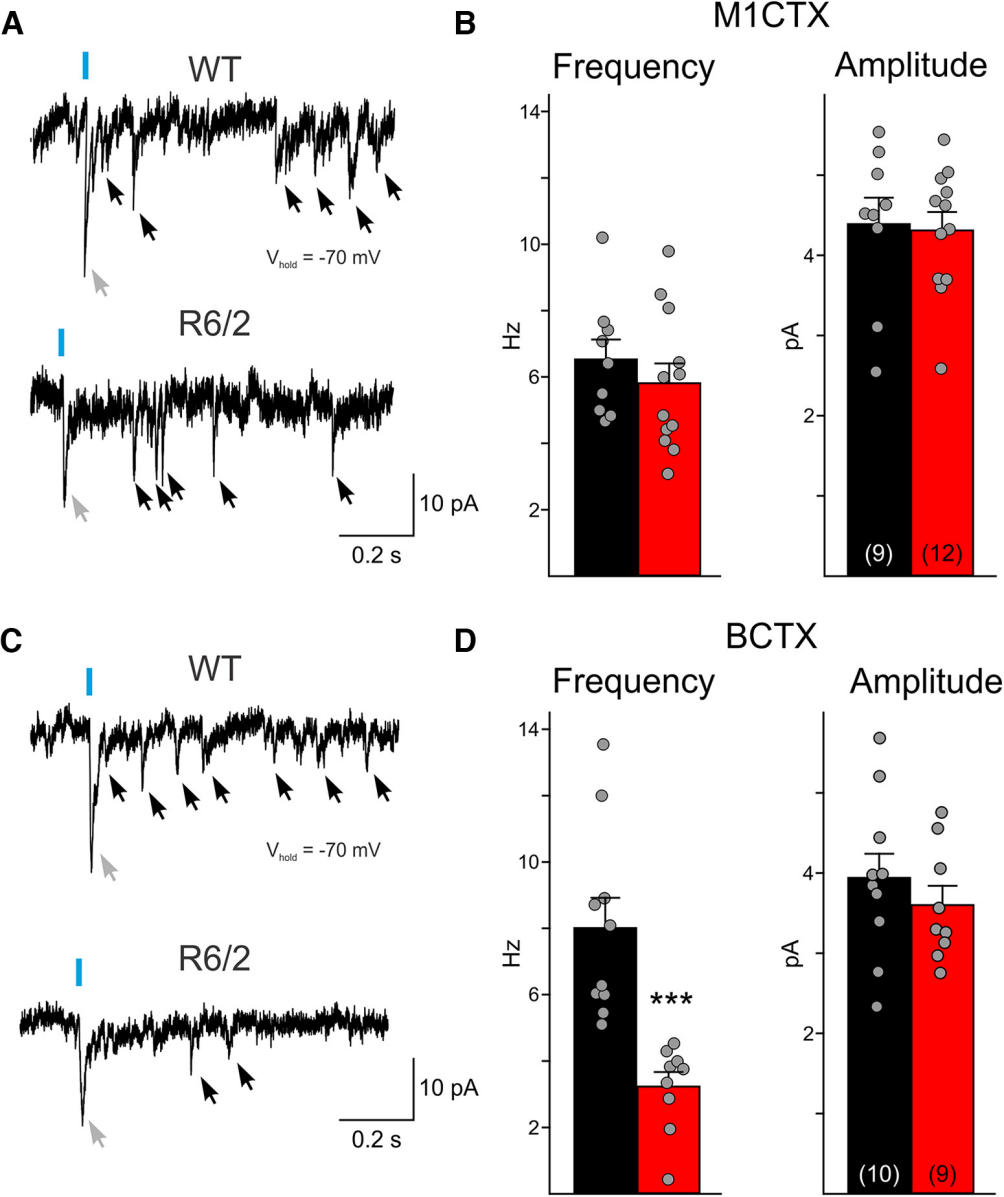
***A***, Sample traces of optically-evoked quantal EPSCs recorded in WT and R6/2 M1CTX CPNs bathed in an ACSF solution where Ca^2+^ was replaced by 4 mm Sr^2+^. Note the large evoked EPSC following blue light is the result of the simultaneous activation of thalamocortical terminals (gray arrow). Single quantal events (black arrows) were measured following the larger evoked current response. ***B***, Summary graphs showing the amplitude and average frequency of quantal events (20 sweeps/cell). ***C***, Sample traces of optically-evoked quantal EPSCs recorded in WT and R6/2 B1CTX CPNs. ***D***, Summary graphs showing the amplitude and average frequency of quantal events (20 sweeps/cell). Statistical significance was determined with Student’s *t* tests; ***p < 0.001.

While thalamus-driven, oEPSCs in cortical CPNs are reduced in R6/2 mice compared with WTs, it is unclear whether these smaller current responses are a result of a decrease in glutamate release from thalamic terminals or whether receptors on CPNs are less responsive to glutamate. To examine alterations in neurotransmitter release, we measured the quantal events elicited following activation of thalamic terminals. We replaced the external concentration of Ca^2+^ with Sr^2+^, a less effective divalent ion that enters into cells through voltage-gated Ca^2+^ channels and is able to bind to vesicular proteins, but favors slow fusion of neurotransmitter-filled vesicles. Upon activation of ChR2 in thalamocortical terminals by blue-light, initial fusion of docked vesicles leads to the small response that is trailed by a series of quantal events (Fig. 7*A*,*C*, black arrows). In M1CTX CPNs, both the amplitude and frequency of quantal events were similar between symptomatic R6/2 and WT CPNs (Fig. 7*B*). However, in BCTX CPNs from R6/2 mice, the frequency of quantal events was significantly reduced (*p* = 0.00026 for R6/2 vs WT), while the amplitudes did not differ (Fig. 7*D*). These findings provide evidence that thalamocortical projections onto CPNs in the BCTX display reduced glutamate release probability in symptomatic R6/2 mice, whereas in the M1CTX other factors could be involved, e.g., reduced number of active release sites.

### AMPA and NMDA receptors respond similarly to glutamate in WT and R6/2 CPNs

**Figure 8.**
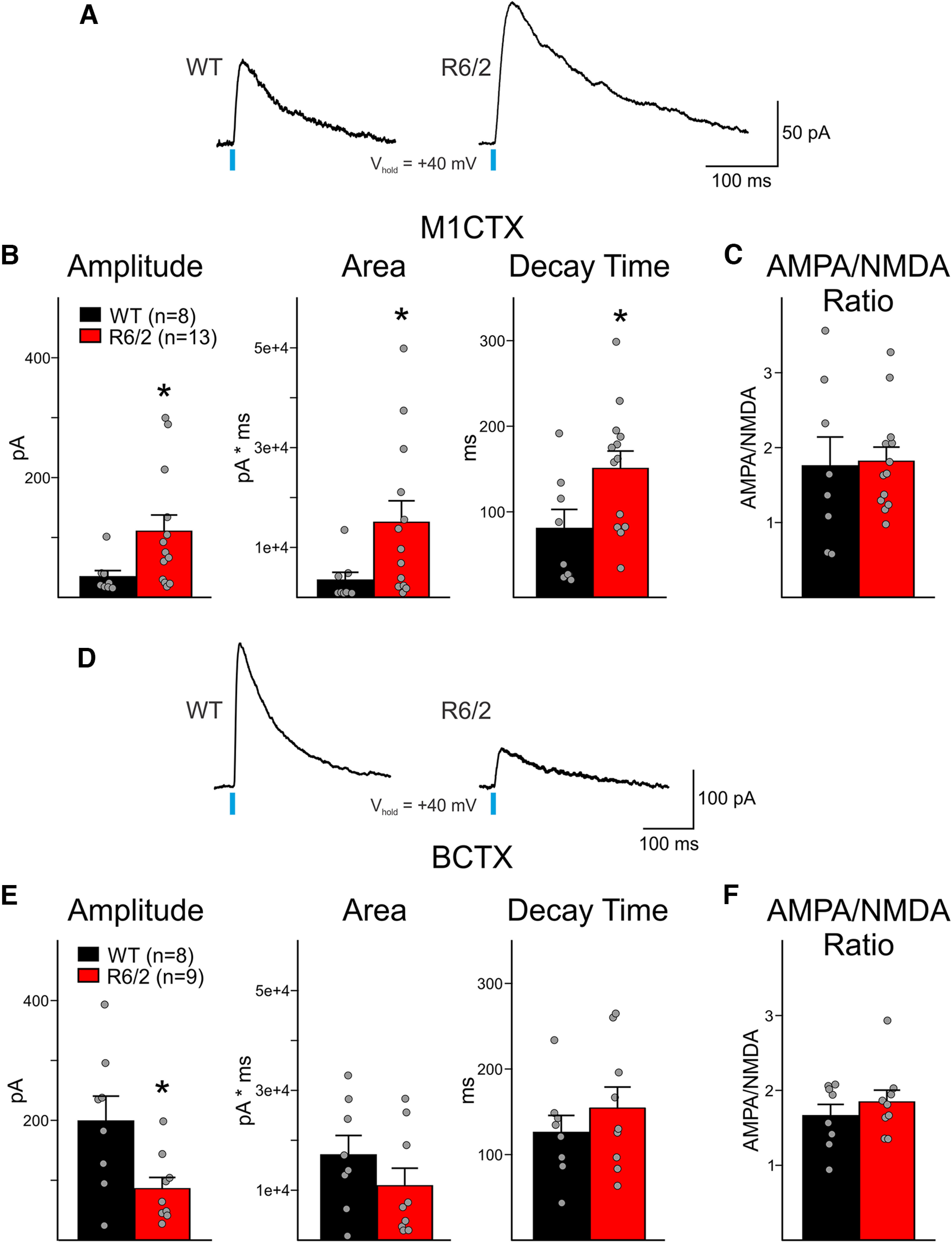
***A***, Sample traces of NMDA-receptor current responses (oNMDA, 3 sweeps, averaged) in WT and R6/2 M1CTX CPNs following optical activation of thalamic inputs (0.5 ms, 470 nm, 3 mW). ***B***, Summary graphs showing optically-evoked NMDA current response properties in M1CTX CPNs. ***C***, AMPA:NMDA current amplitude ratio in M1CTX CPNs. ***D***, Sample traces of NMDA-receptor current responses (oNMDA, 3 sweeps, averaged) in WT and R6/2 BCTX CPNs following optical activation of thalamic inputs (0.5 ms, 470 nm, 3 mW). ***E***, Summary graphs showing optically-evoked NMDA current response properties in BCTX CPNs. ***F***, AMPA:NMDA current amplitude ratio in BCTX CPNs. Statistical significance was determined with Student’s *t* tests or Mann–Whitney rank-sum tests; *p < 0.05.

Since a decrease in receptor sensitivity to glutamate may also explain smaller evoked current responses, we sought out to verify that postsynaptic glutamate receptors respond to glutamate similarly in R6/2 and WT CPNs. In response to optical stimulation of thalamic terminals, slowly-decaying NMDA receptor-currents were produced in CPNs that were held at +40 mV (Fig. 8*A*,*D*). In both M1CTX and BCTX CPNs, optically-evoked NMDA receptor-mediated currents differed between R6/2 and WT mice, albeit differentially. In M1CTX CPNs, the amplitude and area of NMDA receptor-mediated currents were larger in R6/2 CPNs compared with WTs (*p* = 0.012 and *p* = 0.019, respectively; Fig. 8*D*), while in R6/2 BCTX CPNs, current amplitudes were smaller (*p* = 0.0210; Fig. 8*E*). Decay times of NMDA receptor-mediated currents were significantly slower only in M1CTX CPNs in R6/2 mice compared with WTs. In addition, the ratios of AMPA:NMDA current response amplitudes in CPNs for both regions were similar between R6/2 mice and WTs (Fig. 8*C*,*F*), suggesting that CPNs in both areas display similar postsynaptic glutamatergic receptor-mediated responses independent of genotype. Thus, the mechanism underlying the decreased connectivity in R6/2 CPNs observed following optical stimulation of thalamic terminals is most likely because of alterations in presynaptic release.

## Discussion

The present study provides evidence of physiological changes that exist in both the motor and somatosensory thalamocortical pathways in R6/2 mice. We show that some of these alterations can be attributed to a disconnect between cells in the thalamus and cells in their targeted cortical regions. We also observed that cells in both VAL motor and VPM somatosensory thalamic nuclei of symptomatic R6/2 mice were hyperexcitable when compared with WT cells in each region. We also showed that CPNs in both M1CTX and somatosensory BCTX display fewer spontaneous synaptic events, evoked excitatory inputs from the thalamus were significant smaller and, at least for the BCTX, this reduction was related to a decrease in glutamate release from VPM thalamic cells. These data provide considerable evidence that somatosensory and motor thalamic information transfer is disrupted in symptomatic R6/2 mice.

### The HD thalamus

Previous reports in patients and animal models have shown the thalamus exhibits signs of degeneration and atrophy before motor impairments (de la Monte et al., 1988; Kassubek et al., 2005) and more so in advanced symptomatic cases (Dom et al., 1976; Heinsen et al., 1996, 1999). We provide evidence that cells in two thalamic nuclei are altered in R6/2 mice, which ultimately affects their ability to relay salient information to cells in targeted cortical areas. We observed thalamic cells in symptomatic R6/2 mice that project to both motor or somatosensory cortices were more excitable and had altered cell membrane properties when compared with cells in corresponding regions in WT mice. These changes in cell membrane properties (reduced membrane capacitance, increased input resistance and faster time constant) may be attributed to cells undergoing degenerative changes, modifications in cellular morphology and/or significant changes in ion channel distribution. At the younger age, before the presence of overt symptoms, thalamic cells in R6/2 mice did not display increased excitability compared with cells in WTs. However, we observed some changes in cell membrane properties in thalamic cells at the presymptomatic age suggesting that in R6/2 mice, morphologic alterations in thalamic cells either parallels disease progression or begins at a later time. Thalamic cells in R6/2 mice exhibit a progressive decrease in the extracellular matrix (ECM) glycoprotein tenascin-C that may contribute to the cellular atrophy exhibited in this region (Kusakabe et al., 2001). It was shown that tenascin-C was reduced at eight weeks of age and was nearly absent at 12 weeks; and, incidentally, this age coincides with the time when our electrophysiological experiments were performed (range 64–85 d). Along with the decrease in tenascin-C, the R6/2 thalamus shows signs of astrogliosis and disrupted synaptic connectivity (Verkhratsky and Nedergaard, 2014). Astrogliosis is suspected to contribute to neuronal hyperexcitability (Devinsky et al., 2013; Robel and Sontheimer, 2016) by disrupting local GABA:glutamine ratios through the decreased expression of glutamine synthetase. The resulting decrease in GABA production may result in increased neuronal excitability (Khazipov, 2016; Eid et al., 2019; Verhoog et al., 2020). Thus, in addition to the declining expression of tenascin-C, the presence of astrogliosis and increases in excitability indicate thalamic cells in R6/2 mice may progress toward a potentially degenerative state.

VPM cells from R6/2 mice displayed a lower rheobase and a more depolarized RMP. In addition, these cells also received more synaptic inputs as indicated by the observed increase in sPSC frequency. This increase in sPSC frequency also may contribute to the increased excitability observed in these cells, particularly if glutamate is the major synaptic neurotransmitter that is released onto these cells. VPM cells receive extensive glutamatergic inputs particularly from somatosensory pathways, as well as feedback from the cortex and other thalamic nuclei (Jones and Powell, 1968; Guillery, 1995; Sherman and Guillery, 1996; Rovó et al., 2012; Crabtree, 2018). Thus, it is likely that glutamate contributes to the overall increased excitability observed in R6/2 VPM cells, although alterations in inhibitory sources cannot be ruled out.

### The HD cortex

Similar to cells in the thalamus, CPNs in both the motor M1CTX and somatosensory BCTX displayed changes in cell membrane properties (reduced capacitance, increased input resistance, and faster time constants) in symptomatic mice, compared with cells in WT mice. These data were comparable to previously reported data for M1CTX CPNs in R6/2 and Q175 mice (Cummings et al., 2009; Indersmitten et al., 2015). There is considerable evidence that shows the cortex undergoes significant thinning in HD, particularly at grades 2–4 (Mann et al., 1993; Vonsattel and DiFiglia, 1998; Rosas et al., 2005; Hobbs et al., 2010). Symptomatic HD mouse models also exhibit significant cortical atrophy (Gray et al., 2008; Sawiak et al., 2009; Zhang et al., 2010; Cepeda-Prado et al., 2012; Zhang et al., 2020). However, there is very little to no cortical cell loss in the models. Incidentally, it has been reported that R6/2 CPNs and striatal neurons exhibit reduced dendritic spine density compared with WT cells and this loss of spines contributes to the increased membrane input resistance (Klapstein et al., 2001; Murmu et al., 2013). Also, in R6/2 mice, cortical and striatal cells have been shown to reside in a nonapoptotic degenerative state and it has been suggested that although these cells are not labeled with classical apoptosis-dependent cellular markers, they undergo morphologic changes that coincide with the presence of mHTT aggregates (Turmaine et al., 2000). Although it remains unclear whether the presence of mHTT aggregates in cells ultimately leads to neurodegeneration and/or cell death, neurons in several brain regions of symptomatic R6/2 mice that display evidence of morphologic alterations also express mHTT aggregates, albeit to varying degrees (Li et al., 2001). As such, the alterations in cell membrane properties we show in M1 and somatosensory CPNs may be a reflection of underlying degenerative processes.

The decrease in spontaneous synaptic events (sIPSCs and sEPSCs) in CPNs in both R6/2 motor and somatosensory cortices may be because of decreased connectivity between cortical cells and interneurons, and this change is to be expected if these cells are subjected to some sort of degenerative process that progresses with age. These spontaneous synaptic events are a mixture of inputs coming from other local CPNs in addition to GABAergic interneurons, but both areas also receive significant glutamatergic inputs from the thalamus (reviewed in Sherman, 2017). Our optogenetic data suggest that there is a significant decrease in thalamic inputs from both VAL and VPM nuclei to CPNs in their targeted cortical areas. As such, the reduction in spontaneous events may be because of, but not limited to, a diminution in corticothalamic connectivity. It is important to note that in our previous report, CPNs in symptomatic mice displayed an increase in the frequency of sEPSCs (Cummings et al., 2009) while here, we observed a decrease in spontaneous events. This difference is largely because of the absence of large excitatory events that was shown to exist in R6/2 CPNs at later ages, possibly as a rebound effect of diminishing excitatory inputs or in response to the reduction in cortical inhibition. The R6/2 mice in the earlier report had a smaller CAG repeat length (∼111) than the mice used in this study (∼160) that are afflicted with a more aggressive disease progression, and some synaptic changes may not be as readily observable at the age investigated. Nevertheless, based on the earlier study, the frequency of sEPSCs decreased in R6/2 mice with age and symptom severity and likewise, inhibitory inputs were dramatically reduced. These data are in accordance with a decrease in cortical synaptic connections in the late stages of HD. Conversely, previous studies have shown the frequencies of both sIPSCs and sEPSCs were increased in Layers II/III YAC128 barrel cortex CPNs and in CAG140 motor cortex CPNs (Cummings et al., 2009; Sepers et al., 2022). Moreover, motor cortex CPNs in heterozygous Q175 mice also displayed a significant increase in sIPSC frequency compared with WTs while sEPSC frequencies were comparable (Indersmitten et al., 2015). It is also important to note that in these earlier studies, CPNs cell membrane properties also were indistinguishable from those of WT CPNs. This is not surprising, given the differences in the timeline of disease progression and behavioral phenotype between the R6/2 mice and late-onset HD mouse models. R6/2 mice (∼150 CAG repeats) display an aggressive, rapid disease progression that is more akin to the juvenile form of HD. In fact, this model was first developed by introducing an expanded CAG repeat from a patient with juvenile-onset HD into the mouse genome (Mangiarini et al., 1996). It is possible that the cortical pathophysiological abnormalities we observed only apply to this form of HD. Alternatively, the age at which our experiments were performed may correspond to a very late-stage in the disease progression while previous studies performed in 6- to 12-month-old late-onset HD models parallel an earlier stage of the disease when motor and cognitive symptoms are less severe. It would be of interest to see whether similar physiological defects are evident at later ages in late-onset HD models.

One would expect that since we observed thalamic cells in both VAL and VPM nuclei display an increase in excitability that this would lead to an overall increase in glutamatergic inputs onto CPNs. Interestingly, in our optogenetic experiments, we show there is a significant decrease in monosynaptic thalamic inputs onto R6/2 CPNs in both motor and somatosensory barrel cortices compared with WTs. This finding is not surprising because of the observable physiological changes that these CPNs undergo in the late stages of the disease. R6/2 CPNs possess fewer dendritic spines compared with WTs thus equating to a decrease in excitatory inputs. Incidentally, and in agreement with previous reports in R6/2 and BACHD mice (Spampanato et al., 2008; Cummings et al., 2009), inhibitory inputs were reduced, as was evidenced by a decrease in the frequency of sIPSCs. In the cortex, the majority of GABAergic interneurons favor synapsing along dendritic shafts, axons and cell bodies of CPNs, while only a few (∼20%), such as the somatostatin (SOM)-expressing Martinotti cells, synapse directly onto spines (Kwon et al., 2019). A reduction in dendritic spines could contribute to the decrease in sIPSCs, although cortical GABAergic interneurons themselves may also be affected by the disease. In humans, there is a loss of parvalbumin-expressing interneurons in the striatum and cortex of advanced symptomatic HD patients (Thu et al., 2010; Reiner et al., 2013), which would imply that these interneurons undergo cellular changes resulting in a diminution of inhibitory inputs onto CPNs. As such, an imbalance in GABA/glutamate tone would ultimately contribute to the overexcitation of CPNs in response to thalamic inputs, thus resulting in a miscommunication of information within the cortex.

We also observed a significant decrease in the frequency of thalamic-driven quantal release of glutamate at thalamocortical synapses in the somatosensory BCTX of R6/2 mice, when compared with WTs. A change in quantal event frequency, but no change in amplitude is suggestive of presynaptic alterations (Choi and Lovinger, 1997). Interestingly, the amplitude of NMDA receptor-mediated current responses was also decreased in R6/2 somatosensory CPNs when compared with WT CPNs, and yet, we observed no significant differences in AMPA:NMDA amplitude ratios. This would suggest that at these synapses, R6/2 and WT CPNs respond to glutamate similarly, but there is less neurotransmitter being released from thalamic terminals. Thus, despite being more excitable, VPM thalamic cells lack ability to efficiently transmit excitatory information to CPNs. Several factors could affect neurotransmitter release in thalamic cells. Aggregates and toxic nuclear inclusions of mHTT are highly expressed in the cortex as well as in thalamic cells (Morton et al., 2000). Normal HTT interacts directly with synaptic vesicles and assists in the antero- and retrograde trafficking of vesicles in axons (DiFiglia et al., 1995; Saudou and Humbert, 2016) and in R6/2 mice, the presence of mhHTT aggregates impairs vesicular release by disrupting its association with synaptic scaffolding proteins (Li et al., 2003). Considering this, aberrant accumulation of mHTT aggregates in thalamic cell axons may inflict abnormalities in axon structure, cause a shortage of neurotransmitter-containing synaptic vesicles and give rise to deficient release of glutamate at somatosensory thalamocortical synapses. Since we did not observe the same decrease in quantal release probability at R6/2 M1CTX thalamocortical synapses, the decrease in optically-evoked AMPA currents along with a significant decrease in mEPSC frequency would suggest that, although presynaptic release of glutamate may be affected in this cortical region in symptomatic R6/2 mice, this does not completely rule out that other potential mechanisms could be involved in M1CTX CPNs. Given that cell membrane capacitance is smaller and a loss of dendritic spines was previously reported in M1CTX CPNs (Klapstein et al., 2001), it is plausible that the number of active glutamate receptors and release sites may actually be reduced in these cells. Although progressive spine loss has previously been observed in somatosensory cortex CPNs (Murmu et al., 2013), our data suggest presynaptic alterations as the major cause of reduced thalamocortical connectivity in the R6/2 BCTX.

Although there were no significant genotype-dependent differences in M1CTX or BCTX AMPA:NMDA ratios in the CPNs of symptomatic R6/2 mice, we did observe contrasting alterations in the optically-evoked NMDA receptor-mediated current responses between the two brain regions. Compared with WTs, optically-evoked NMDA-mediated current responses in R6/2 M1CTX CPNs were larger in magnitude (larger amplitude, greater area and longer decay time) while the amplitude of evoked currents in BCTX CPNs were smaller. Differential expression of NMDA receptors in each brain area or within each specific cortical layer may explain these differences. A previous study in healthy mice suggested there is a greater density of NMDA receptors in the somatosensory cortex than in the motor cortex since NMDA receptor-dependent long-term potentiation (LTP) is more easily induced in the former (Castro-Alamancos and Connors, 1996). In HD mice, mHTT inclusions were reported to occlude the interaction of NMDA receptors with membrane scaffolding proteins (Sun et al., 2001). Despite a possible higher expression of NMDA receptors, evoked NMDA receptor-mediated current responses in R6/2 BCTX CPNs would appear smaller because of the increased presence of mHTT inclusions in this cortical area than in the motor cortex (Cabanas et al., 2020). In addition, mHTT can cause the switching to alternative forms or the reorganization of postsynaptic density proteins (PSDs) associated with NMDA receptor-rich synapses and may lead to the disruption of glutamatergic transmission (Torres-Peraza et al., 2008). Taking this into account, our data suggest that NMDA receptors in R6/2 M1CTX CPNs are likely to undergo changes that differ from what is seen in the BCTX and that would account for larger evoked current responses that we report here. Further investigations comparing NMDA receptor subunit expression, receptor distribution and changes in membrane scaffolding complexes within different cortical regions in HD models are needed to explain the differences seen here and to better our understanding of the sensory and cognitive disturbances observed in patients afflicted with the disease.

### Thalamocortical connectivity in HD

We provide considerable evidence that thalamic and cortical cells involved in the integration and processing of sensory and motor information as well as the planning and execution of movements are impaired in symptomatic R6/2 mice. This evidence shows there is a disruption in the functional connectivity between cells in the thalamus and cells in their targeted cortical areas. This disconnect would ultimately cause a delay in the initiation and maintenance of motor activity. Our data showing reduced thalamic input to the somatosensory cortex could explain deficits and delays in sensory responsiveness that is observed in HD patients (Fellows et al., 1997; Boecker et al., 1999). Reduced thalamic input to other cortical areas could also explain deficits in visual discrimination (Brouwers et al., 1984) and the presence of auditory hallucinations (Albin and Young, 1988). It was recently shown in Q175 HD mice that thalamocortical coherence is altered and neuronal firing in response to an auditory stimuli was delayed (Shobe et al., 2021). This study also reported a significant delay in firing of fast-spiking interneurons in the cortex when confronted with a behavioral cue. We observed that evoked thalamus-driven inhibitory responses were smaller in both the motor and somatosensory barrel cortex CPNs, suggesting that local cortical inhibition may also be disturbed as a result of defective thalamic input. In addition to fast-spiking interneurons, SOM-expressing and vasoactive intestinal peptide (VIP)-expressing interneurons also receive thalamic inputs and these interneurons assist in priming CPNs to respond to salient information in addition to establishing cortical inhibitory tone. Needless to say, more studies are warranted to further dissect how cortical inhibition is disrupted and what specific interneuron populations are most susceptible in HD.

## Synthesis

Reviewing Editor: Laura Volpicelli-Daley, The University of Alabama at Birmingham Decisions are customarily a result of the Reviewing Editor and the peer reviewers coming together and discussing their recommendations until a consensus is reached. When revisions are invited, a fact-based synthesis statement explaining their decision and outlining what is needed to prepare a revision will be listed below. The following reviewer(s) agreed to reveal their identity: Lynn Raymond.

To summarize, both reviewers felt this is an important study that was well executed. The main request is to include images showing the labeling with the GFP retrobeads: “Since the identification of the neurons in thalamus, that project to the different cortical regions depends on the labeling by GFP-labeled retrobeads injected into the cortex, the authors need to provide images demonstrating the robustness of this approach. “

Most of the concerns can be addressed in writing, particularly specifying which cortical layers are being recorded.

Reviewer 1:

Using electrophysiology and optogenetics in acute brain slice, the authors examine the function of thalamic neurons in VPM and VAL that project to barrel cortex and motor cortex, respectively, in the R62 mouse model of Huntington disease. They show that the thalamic neurons that project to M1 from VAL and to barrel cortex from VPM show increased excitability, but reduced synaptic EPSCs. Stimulating these neurons optogenetically after injecting channelrhodopsin reveals reduced amplitude of oIPSCs and oEPSCs recorded fom CPNs. They then do a clever experiment using optogenetic stimulation and extracellular strontium to measure spontaneous vesicular transmitter release from thalamic terminals onto CPNs; they found a decreased frequency of events from terminals of VPM onto barrel cortex CPNs. They also showed that the AMPA/NMDA ratio of oEPSCs recorded from M1 cortex and barrel cortex were similar between genotypes, suggesting similar responsiveness of receptors, although isolated NMDA receptor-mediated responses show larger amplitude in M1 cortex and smaller amplitude in barrel cortex.

Altogether, these results are novel and significant for the field, in that it the thalamus also shows significant degeneration in Huntington disease, but when this occurs and how it functionally affects thalamic connections to cortex has not been elucidated previously. This is a very important connection because of its key role in sensory motor processing and motor programming. Results of this study should be of wide interest to the field.

I have a few minor comments:

1) In the Abstract the authors state that they did patch clamp recording from VAL and VPM thalamic neurons in slice to determine membrane properties of these neurons; however, in the Introduction, page 4, first paragraph, lines 2 through 5, they indicated they made patch clamp recording of “cell membrane properties and synaptic inputs onto CPNs...”. This suggests that patch clamp recordings are all from CPNs in the cortex and none from thalamic neurons. In addition, second to last sentence in the Introduction reiterates “we report that passive and active cell membrane properties of CPNs...”. The authors should clarify the wording here to indicate they make recordings from both CPNs and thalamic neurons, both in the Abstract and the Introduction.

2) In the Methods, page 7: where in the cortex were CPNs recorded from - which layer? In the Results section, bottom of Page 12, there is still no indication of which layer the CPNs are recorded from. On bottom of page 13, when recording oIPSCs from VAL and VPM onto CPNs they do mention layer 2 in M1 cortex, although there is still no mention of which layer in barrel cortex. Also, there is no mention of the cortical layer they record from for oEPSCs. Please clarify the cortical layer(s) in both Methods and Results.

3) Page 15, the last sentence of first paragraph. Please remove the phrase “synaptic glutamate release from” and just leave it as “thalamic cortical projections onto CPNs in the barrel cortex... display reduced glutamate release”.

4) The authors’ conclusion that the mechanism of reduced connectivity from thalamus to CPNs in R62 mice is due to reduced presynaptic glutamate release seems reasonable for the connection of VPM to barrel cortex (decreased frequency of quantal release, unchanged AMPA/NMDA ratio when NMDAR EPSCs are smaller). However, for VAL to M1 cortex, the normal AMPA/NMDA ratio in the context of larger NMDAR currents suggests increased glutamate release at activated synapses (since synaptic AMPARs are not saturated by normal levels of glutamate release whereas NMDARs are). Together, with the reduced oEPSCs onto the CPNs from VAL suggest there may be reduced numbers of synapses.

5) Legend for Figure 5B and D: please change “IPSC” to “EPSC"

Reviewer 2:

The mutant Huntingtin protein is found in cells throughout the brain. However, most of the work in Huntington’s disease (HD) has focused on the changes present in the caudate nucleus and putamen (striatum) as this is the most degenerated region in HD patients. Furthermore, the clinical manifestations of HD can be easily attributed to the degeneration of the medium spiny neurons (MSNs) in the striatum which are critical for proper motor activities.

There have been many studies performed in the mouse models expressing various forms of mutant Huntingtin (mHTT) to assess electrophysiological deficits in the MSNs and in cortical projection neurons. This manuscript is focused on studying two different thalamic nuclei; somatosensory (VPM) and motor (VPL) nuclei and their targets in the motor cortex (M1 cortex) and the somatosensory barrel (BC cortex) cortex respectively. The thalamus is a critical component of the basal ganglia circuit and is a very understudied area in HD. Thus, understanding how cells innervated by neurons from these two nuclei contribute to the function of the cortical pyramidal neurons in HD is an area in need of study. The authors used the R6/2 mHTT expressing mouse model to demonstrate electrophysiological alterations in the specific thalamic nuclei neurons and in the cortical pyramidal neurons to which they project.

Overall, the presented work is well done. However, there are a few additions that would improve the manuscript.

1. Care must be taken in describing “degeneration” in the R6/2 mouse (as on page 4). Given that there is minimal overt neuronal cell death in the model, it must be clear what the authors mean by degeneration.

2. The authors seem to equate the presence of aggregated mhtt in R6/2 mice as support for the degeneration across different brain regions and thus the different brain regions’ contribution to HD symptomatology. This is not necessarily the case. The presence of aggregates in a specific region does not necessarily mean that there is degeneration in that region. Many of the mouse model expressing mutant Huntingtin contain numerous aggregates throughout the brain without significant evidence of degeneration. This must be made clear in the writing.

3. When discussing the degeneration of thalamic nuclei in the introduction, the authors need to be specific about which nuclei they are describing. Do those nuclei include those under investigation in this manuscript?

4. Since the identification of the neurons in thalamus, that project to the different cortical regions depends on the labeling by GFP-labeled retrobeads injected into the cortex, the authors need to provide images demonstrating the robustness of this approach.

5. Additionally, a figure needs to be provided that also demonstrates the robustness of the optogenetic paradigm used to activate the VAL and VPM neurons.

6. The CPN cells in the R6/2 mice display regional differences in membrane properties, did the authors evaluate the size or morphology of the neurons recorded from?

7. In the discussion the authors should compare the results observed for the other mouse models expressing mHTT that performed studies in the barrel and sensory cortices. Are the obtained results in the manuscript in line with what has been observed in the various other models?

8. One must be careful when discussing the GABA:glutamine ratio as being altered by astrocytes in the R6/2 model. In addition to the electrophysiological changes, is there more evidence of altered extracellular GABA or glutamate levels in the R6/2 mouse model in the regions studied here?

9. Please discuss how the findings presented here for study of the motor and somatosensory cortex in the R6/2 mouse model compare to results obtained in other models.

10. Please discuss results in context of the behavioral manifestations observed in the R6/2 mice at the age of the recordings. Furthermore, how do the changes observed for these thalamic nuclei relate to the clinical presentations observed in HD patients.

## Author Response

We thank the editor and reviewers for their kind and supportive remarks. Below is a point-by-point reply to the minor concerns raised by both reviewers.

Editor’s Synthesis Statement for Author:

To summarize, both reviewers felt this is an important study that was well executed. The main request is to include images showing the labeling with the GFP retrobeads: “Since the identification of the neurons in thalamus, that project to the different cortical regions depends on the labeling by GFP-labeled retrobeads injected into the cortex, the authors need to provide images demonstrating the robustness of this approach. “

Most of the concerns can be addressed in writing, particularly specifying which cortical layers are being recorded.

Response: A new figure demonstrating the utility and robustness of the retrobead identification of thalamic cells is now included in the manuscript (Fig. 1).

Reviewer 1:

Using electrophysiology and optogenetics in acute brain slice, the authors examine the function of thalamic neurons in VPM and VAL that project to barrel cortex and motor cortex, respectively, in the R62 mouse model of Huntington disease. They show that the thalamic neurons that project to M1 from VAL and to barrel cortex from VPM show increased excitability, but reduced synaptic EPSCs. Stimulating these neurons optogenetically after injecting channelrhodopsin reveals reduced amplitude of oIPSCs and oEPSCs recorded fom CPNs. They then do a clever experiment using optogenetic stimulation and extracellular strontium to measure spontaneous vesicular transmitter release from thalamic terminals onto CPNs; they found a decreased frequency of events from terminals of VPM onto barrel cortex CPNs. They also showed that the AMPA/NMDA ratio of oEPSCs recorded from M1 cortex and barrel cortex were similar between genotypes, suggesting similar responsiveness of receptors, although isolated NMDA receptor-mediated responses show larger amplitude in M1 cortex and smaller amplitude in barrel cortex.

Altogether, these results are novel and significant for the field, in that it the thalamus also shows significant degeneration in Huntington disease, but when this occurs and how it functionally affects thalamic connections to cortex has not been elucidated previously. This is a very important connection because of its key role in sensory motor processing and motor programming. Results of this study should be of wide interest to the field. I have a few minor comments:

1) In the Abstract the authors state that they did patch clamp recording from VAL and VPM thalamic neurons in slice to determine membrane properties of these neurons; however, in the Introduction, page 4, first paragraph, lines 2 through 5, they indicated they made patch clamp recording of “cell membrane properties and synaptic inputs onto CPNs...”. This suggests that patch clamp recordings are all from CPNs in the cortex and none from thalamic neurons. In addition, second to last sentence in the Introduction reiterates “we report that passive and active cell membrane properties of CPNs...”. The authors should clarify the wording here to indicate they make recordings from both CPNs and thalamic neurons, both in the Abstract and the Introduction.

Response: A sentence was added at the end of the Introduction to clarify that in addition to recording from CPNs, recordings were performed in thalamic cells.

2) In the Methods, page 7: where in the cortex were CPNs recorded from - which layer? In the Results section, bottom of Page 12, there is still no indication of which layer the CPNs are recorded from. On bottom of page 13, when recording oIPSCs from VAL and VPM onto CPNs they do mention layer 2 in M1 cortex, although there is still no mention of which layer in barrel cortex. Also, there is no mention of the cortical layer they record from for oEPSCs. Please clarify the cortical layer(s) in both Methods and Results.

Response: The cortical layers from which electrophysiological recordings were performed is now indicated in both the Methods and Results. In addition, we specified that in MCTX, recordings were obtained in layers II/III CPNs, since individual layers II and III are undistinguishable in murine coronal brain slices.

3) Page 15, the last sentence of first paragraph. Please remove the phrase “synaptic glutamate release from” and just leave it as “thalamic cortical projections onto CPNs in the barrel cortex... display reduced glutamate release”.

Response: As suggested by the reviewer, the phrase was removed.

4) The authors’ conclusion that the mechanism of reduced connectivity from thalamus to CPNs in R62 mice is due to reduced presynaptic glutamate release seems reasonable for the connection of VPM to barrel cortex (decreased frequency of quantal release, unchanged AMPA/NMDA ratio when NMDAR EPSCs are smaller). However, for VAL to M1 cortex, the normal AMPA/NMDA ratio in the context of larger NMDAR currents suggests increased glutamate release at activated synapses (since synaptic AMPARs are not saturated by normal levels of glutamate release whereas NMDARs are). Together, with the reduced oEPSCs onto the CPNs from VAL suggest there may be reduced numbers of synapses.

Response: We agree with the reviewer that our conclusion that the reduction in VAL-M1CTX connectivity is difficult to explain by purely presynaptic mechanisms. Although reduced frequency of mEPSCs in M1 CPNs is suggestive of decreased release probability, the fact that quantal release measured in the presence of Sr2+ only displayed a small trend for reduced glutamate release implies that other mechanisms may be involved. Thus, the possibility of a reduced number of synapses cannot be ruled out. Indeed, reduced number of dendritic spines in CPNs would support this conclusion. These points are now elaborated in the Discussion.

5) Legend for Figure 5B and D: please change “IPSC” to “EPSC"

Response: We thank the reviewer for catching this. The change was made.

Reviewer 2:

The mutant Huntingtin protein is found in cells throughout the brain. However, most of the work in Huntington’s disease (HD) has focused on the changes present in the caudate nucleus and putamen (striatum) as this is the most degenerated region in HD patients. Furthermore, the clinical manifestations of HD can be easily attributed to the degeneration of the medium spiny neurons (MSNs) in the striatum which are critical for proper motor activities.

There have been many studies performed in the mouse models expressing various forms of mutant Huntingtin (mHTT) to assess electrophysiological deficits in the MSNs and in cortical projection neurons. This manuscript is focused on studying two different thalamic nuclei; somatosensory (VPM) and motor (VPL) nuclei and their targets in the motor cortex (M1 cortex) and the somatosensory barrel (BC cortex) cortex respectively. The thalamus is a critical component of the basal ganglia circuit and is a very understudied area in HD. Thus, understanding how cells innervated by neurons from these two nuclei contribute to the function of the cortical pyramidal neurons in HD is an area in need of study. The authors used the R6/2 mHTT expressing mouse model to demonstrate electrophysiological alterations in the specific thalamic nuclei neurons and in the cortical pyramidal neurons to which they project.

Overall, the presented work is well done. However, there are a few additions that would improve the manuscript.

1. Care must be taken in describing “degeneration” in the R6/2 mouse (as on page 4). Given that there is minimal overt neuronal cell death in the model, it must be clear what the authors mean by degeneration.

Response: In an effort to avoid confusion, we removed the term “degeneration” and clarified by summarizing the physiological changes that we observed.

2. The authors seem to equate the presence of aggregated mhtt in R6/2 mice as support for the degeneration across different brain regions and thus the different brain regions’ contribution to HD symptomatology. This is not necessarily the case. The presence of aggregates in a specific region does not necessarily mean that there is degeneration in that region. Many of the mouse model expressing mutant Huntingtin contain numerous aggregates throughout the brain without significant evidence of degeneration. This must be made clear in the writing.

Response: We included some clarifications in the Discussion (HD Cortex section). We did not mean to imply that the presence of mHTT aggregates leads to degeneration but that in R6/2 mice brain regions, particularly the cortex, where there are visible signs of atrophy, mHTT aggregates are present. We emphasize that it remains unclear whether or not the presence of aggregates leads to cellular degeneration.

3. When discussing the degeneration of thalamic nuclei in the introduction, the authors need to be specific about which nuclei they are describing. Do those nuclei include those under investigation in this manuscript?

Response: Specific thalamic nuclei that exhibit signs of degeneration are now described in the Introduction. These include mediodorsal (MD), centromedian/parafascicular (CM/Pf), intralaminar (IL), VAL and VPM nuclei.

4. Since the identification of the neurons in thalamus, that project to the different cortical regions depends on the labeling by GFP-labeled retrobeads injected into the cortex, the authors need to provide images demonstrating the robustness of this approach.

Response: A new figure was added that illustrates the utility and robustness of retrobead-labelling of thalamic cells in specific nuclei (Fig. 1A&1B). In addition, the fact that enough cells for recordings were identified in the corresponding thalamic nuclei further supports the robustness of the approach.

5. Additionally, a figure needs to be provided that also demonstrates the robustness of the optogenetic paradigm used to activate the VAL and VPM neurons.

Response: Included in the new Fig. 1 are images and sample recorded traces that assist in demonstrating the robustness of the optogenetic paradigm. In fact, all optical stimulations evoked responses in CPNs and their amplitudes were large prior to the addition of antagonists and TTX.

6. The CPN cells in the R6/2 mice display regional differences in membrane properties, did the authors evaluate the size or morphology of the neurons recorded from?

Response: Although we had every intention of quantifying and evaluating morphological changes in recorded cells this was not done due to the limited number of cells that remained fully intact and undamaged following recordings and histochemical processing. However, examples of biocytin-filled cells are included in the new Fig. 1.

7. In the discussion the authors should compare the results observed for the other mouse models expressing mHTT that performed studies in the barrel and sensory cortices. Are the obtained results in the manuscript in line with what has been observed in the various other models?

Response: We expanded the Discussion to include comparisons between our findings and previous studies performed in late-onset HD models.

8. One must be careful when discussing the GABA:glutamine ratio as being altered by astrocytes in the R6/2 model. In addition to the electrophysiological changes, is there more evidence of altered extracellular GABA or glutamate levels in the R6/2 mouse model in the regions studied here?

Response: There have been very few studies examining extracellular levels of glutamate and/or GABA in the cortex of R6/2 mice and reports of the neurotransmitter levels in the thalamus to our knowledge do not exist. In addition, the methodologies used to measure baseline levels of neurotransmitters (HPLC, in situ hybridization, microdialysis, MRI) differ between groups and results have varied. In the Discussion, we provide a plausible mechanism for how the presence of astrogliosis in R6/2 mice may contribute to an increase in hyperexcitability in the thalamus, although more definitive studies in this region are necessary.

9. Please discuss how the findings presented here for study of the motor and somatosensory cortex in the R6/2 mouse model compare to results obtained in other models.

Response: In the Discussion, we introduced several comparisons of our data with published reports that investigated cortical alterations in Q175, YAC128, CAG140 and BACHD model mice.

10. Please discuss results in context of the behavioral manifestations observed in the R6/2 mice at the age of the recordings. Furthermore, how do the changes observed for these thalamic nuclei relate to the clinical presentations observed in HD patients.

Response: Typical behavioral symptoms observed in R6/2 mice at the age of recordings are now listed at the beginning of the Results section. We also included in the Discussion (Thalamocortical connectivity in HD) some clinical findings observed in HD patients that may be explained by our data recorded in R6/2 mice.
